# Retrieval of the Alzheimer's amyloid precursor protein from the endosome to the TGN is S655 phosphorylation state-dependent and retromer-mediated

**DOI:** 10.1186/1750-1326-5-40

**Published:** 2010-10-11

**Authors:** Sandra I Vieira, Sandra Rebelo, Hermann Esselmann, Jens Wiltfang, James Lah, Rachel Lane, Scott A Small, Sam Gandy, Edgar F da Cruz e Silva, Odete AB da Cruz e Silva

**Affiliations:** 1 Neuroscience, Centre for Cell Biology, Health Sciences Department SACS, University of Aveiro, Aveiro 3810, Portugal; 2 Department of Psychiatry and Psychotherapy, Rhine State Hospital, University of Duisburg-Essen, Germany; 3 Emory University Atlanta, Emory Cognitive Neurology Program, Atlanta, GA 30329, USA; 4 Mount Sinai School of Medicine, New York, NY 10029, USA; 5 Taub Institute for Research on Alzheimer's Disease and the Aging Brain, Department of Neurology, Columbia University College of Physicians and Surgeons, New York, NY 10032, USA; 6 Signal Transduction Laboratories, Centre for Cell Biology, Health Sciences Department SACS, University of Aveiro, Aveiro 3810, Portugal

## Abstract

**Background:**

Retrograde transport of several transmembrane proteins from endosomes to the trans-Golgi network (TGN) occurs via Rab 5-containing endosomes, mediated by clathrin and the recently characterized retromer complex. This complex and one of its putative sorting receptor components, SorLA, were reported to be associated to late onset Alzheimer's disease (AD). The pathogenesis of this neurodegenerative disorder is still elusive, although accumulation of amyloidogenic Abeta is a hallmark. This peptide is generated from the sucessive β- and γ- secretase proteolysis of the Alzheimer's amyloid precursor protein (APP), events which are associated with endocytic pathway compartments. Therefore, APP targeting and time of residence in endosomes would be predicted to modulate Abeta levels. However, the formation of an APP- and retromer-containing protein complex with potential functions in retrieval of APP from the endosome to the TGN had, to date, not been demonstrated directly. Further, the motif(s) in APP that regulate its sorting to the TGN have not been characterized.

**Results:**

Through the use of APP-GFP constructs, we show that APP containing endocytic vesicles targeted for the TGN, are also immunoreactive for clathrin-, Rab 5- and VPS35. Further, they frequently generate protruding tubules near the TGN, supporting an association with a retromer-mediated pathway. Importantly, we show for the first time, that mimicking APP phosphorylation at S655, within the APP 653YTSI656 basolateral motif, enhances APP retrieval via a retromer-mediated process. The phosphomimetic APP S655E displays decreased APP lysosomal targeting, enhanced mature half-life, and decreased tendency towards Abeta production. VPS35 downregulation impairs the phosphorylation dependent APP retrieval to the TGN, and decreases APP half-life.

**Conclusions:**

We reported for the first time the importance of APP phosphorylation on S655 in regulating its retromer-mediated sorting to the TGN or lysosomes. Significantly, the data are consistent with known interactions involving the retromer, SorLA and APP. Further, these findings add to our understanding of APP targeting and potentially contribute to our knowledge of sporadic AD pathogenesis representing putative new targets for AD therapeutic strategies.

## Background

Alzheimer's disease (AD) is a multifactorial disorder, with various contributing factors including genetic predisposition and anomalous protein trafficking [1-4]. All AD forms present characteristic extracellular amyloid plaques, whose main protein constituent is the 4 kD amyloidogenic Abeta (reviewed in [5]). This peptide is generated by two consecutive proteolytic cleavages of its precursor, the Alzheimer's amyloid precursor protein (APP), and is constitutively produced and secreted at low levels during APP trafficking [6,7].

APP traffic is tightly regulated and the protein is cleaved by specific proteases. APP follows the constitutive secretory pathway, being N-glycosylated in the endoplasmic reticulum (ER) and further O-glycosylated (maturation) in the Golgi, where it is highly abundant. APP can be packaged into secretory vesicles in the trans Golgi network (TGN) and delivered to the plasma membrane (PM). Cell surface APP may be cleaved to sAPP or reinternalized into the endocytic pathway [4,8,9]. During this trafficking, full length APP is cleaved to proteolytic fragments including sAPP (soluble APPα, soluble APPβ), Abeta, p3, and the APP intracellular C-terminal domain (AICD), with physiological and/or pathological relevance [10]. The initial cleavage of APP is executed either by α- (ADAM 10 and/or 17) [11] or β-secretase (BACE-1) [12], producing α- or β-soluble APP (α/βsAPP), respectively, and a membrane-bound C-terminal fragment (α/βCTF) (reviewed in [10]). CTFs undergo further cleavages by the γ-secretase complex to produce p3 (from αCTF) or Abeta (from βCTF), along with the ~50 amino acid AICD fragment [13,14]. The majority of Abeta production is believed to occur at the TGN and endocytic vesicles [2,15,16] and dysfunctions in the endosomal-lysosomal pathways have been reported in AD and are likely to be associated with AD pathology [17-20].

Sorting and targeting of APP upon endocytosis appears to be critically important in Abeta production and in AD etiology. In fact, the decreased levels and polymorphisms (particularly those exhibiting lower expression levels) of the APP-binding and sorting protein SorLA were associated with AD and mild cognitive impairment [21-23]. Decreased SorLA levels were associated with sporadic but not familial AD [24]. The neuron-enriched SorLA belongs to the mammalian family of vacuolar protein sorting 10 (VPS10)-containing proteins [25] and, like Sortilin (another member of this family), acts as a retromer-binding receptor [21,26,27].

The retromer is a multi-subunit complex that regulates endosome-to-TGN sorting and transport of transmembranar proteins, such as the mannose 6-phosphate receptor (MPR) in mammals and VPS10 in *S. cerevisiae *[[28], reviewed in [29,30]]. The endosomal-to-TGN retrieval of several proteins was recently found to involve the retromer complex [31] and intermediate clathrin-coated endocytic vesicles that carry the early endosomal Rab5 marker [32,33]. A late step of retromer-dependent vesicular tubulation is necessary for the sorting of proteins away from endosomes. The retromer complex consists of two sorting nexin subunits and a cargo-recognition trimer (VPS26, VPS29, VPS35) [reviewed in [34,35]]. Several findings indicate that dysfunctional retromer complexes can be related to AD pathology, with the components VPS35 and VPS36, being found deficient in sporadic AD brains [reviewed in [28,36,37]]. Further, as for SorLA, modulation of the retromer components inversely correlates with Abeta levels [36,37].

Recently some authors have hypothesized that these correlations most probably occur via retromer and SorLA-dependent APP recycling between the endocytic compartment and the TGN [4]. Although strong experimental evidence already supports a role for SorLA in APP recycling and APP processing [21,27,38,39], retromer-dependent APP retrograde traffic from the endosome to the TGN has not been directly demonstrated to date. In the work here described, we address APP signals that mediate its TGN retrieval in order to better characterize this trafficking route. Clues are evident in the retromer-mediated Golgi retrieval of Sortilin, CIMPR (cation-independent mannose 6-phosphate receptor) and SMAP2 (an ARF GTPase-activating protein) proteins. Trafficking of the latter was shown to involve the clathrin AP-1 adaptor and an YXXϕ targeting signal in the cytoplasmic tail of the cargo [40-42]. The latter is a known basolateral sorting signal (where × is any residue, and ϕ is an aliphatic Leu or Ile or an aromatic amino acid). AP-1 and YXXϕ have been related to protein traffic between endosomes and TGN, in both directions. APP possesses such an YXXϕ sorting signal, ^653^YTSI^656 ^(human APP_695 _isoform numbering), responsible for AP-1 binding and mediating APP basolateral sorting [43]. Phosphorylation within this sorting motif appears to modulate this trafficking, and we have recently reported that mimicking phosphorylation at the serine 655 residue (S655, APP_695 _numbering) enhances APP secretory traffic and increases sAPP production by the alpha-secretase pathway [44]. In the present manuscript, we describe the endosome-TGN recycling pathway taken by APP. All the results are consistent with a model of retromer-mediated APP retrieval to the TGN, which is enhanced by direct APP phosphorylation at its cytoplasmic S655 residue.

## Results

### Endocytosed APP is recycled to the TGN and sorted into tubular structures

**Figure 1:**
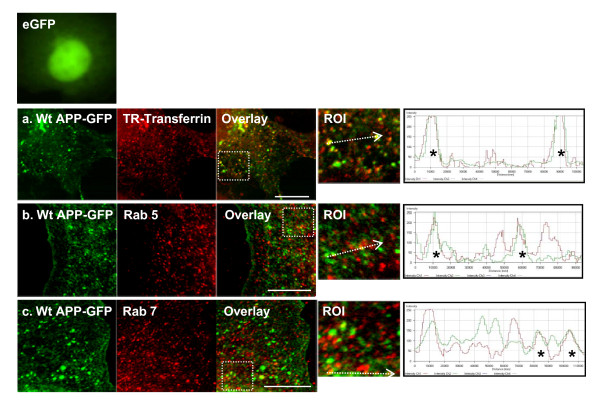
**APP-GFP traverses the endocytic pathway**. **(a) **COS-7 cells transfected with Wt APP-GFP were exposed for 3 h to the protein synthesis inhibitor CHX and incubated in the last 15 min with Texas Red-conjugated transferring. Co-localization (yellow/orange fluorescence vesicles) between transferrin (Texas red endocytic vesicles) and APP-GFP green cytoplasmic vesicles can be observed (Overlay panel). Immunocytochemistry analysis with **(b) **an early (Rab 5) and **(c) **late (Rab 7) endosomal markers were also performed and co-localization observed. ROI, region of interest, denotes APP-GFP co-localizing endocytic vesicles. Bar, 10 μm. Fluorescence intensity profiles are also presented, representing the voxels through the white arrowed lines indicated in the ROI overlay images; asterisks denote co-localizing vesicles. A microphotograph of the eGFP protein is presented as a negative control, GFP alone is distributed through the nucleus and cytoplasm, but is not targeted to cytoplasmic vesicles.

COS-7 cells transfected with Wt APP-GFP at expression levels similar to endogenous APP, were treated with cycloheximide (CHX) for 3 h, thus blocking "de novo" protein synthesis and permitting us to monitor turnover of a specific APP-GFP population [45,46]. APP-GFP endocytosis was analyzed by co-localization with three proteins that are associated with the endocytic route (Fig. 1). In the first panel (Fig. 1a), Texas red-conjugated transferrin molecules were added to the medium, and endocytosis was allowed to occur for 15 min at 37°C. A high number of APP-GFP green vesicles could be observed throughout the cytoplasm of transfected cells, part of this population co-localizes with transferrin-positive vesicles (yellow/orange vesicles, Fig. 1a ROI), confirming the co-endocytosis of APP-GFP together with transferrin. A subset of the visible APP-GFP vesicular population is therefore travelling via the endocytic pathway. This was further confirmed by co-localization of APP-GFP with Rab5 and Rab7 by immunocytochemistry procedures (Fig. 1b and 1c). These are two small GTPases of the Rab family that are associated with early and late endosomal vesicles, respectively. A significant number of APP-GFP/Rab5 and APP-GFP/Rab7 endocytic vesicles could also be detected (Fig. 1b and 1c, respectively, yellow/orange vesicles in ROIs, asterisks in the histograms). Quantitative analyses, using the Zeiss confocal software, were performed, and co-localization of endocytic markers and APP was confirmed (Table 1). Of note, the eGFP protein alone is distributed through the nucleus and cytoplasm, but is not targeted to cytoplasmic vesicles [47].

**Table 1:** Co-localization of the Wt APP-GFP protein with the endocytic markers Transferrin, Rab 5, and Rab 7.

	Wt APP-GFP co-localization coefficients (%)	
	**In cytoplasmic vesicles**	**In cell (cytoplasmic vesicles and Golgi)**

Transferrin	18.8 ± 0.3	34.5 ± 2.4

Rab 5	19.5 ± 0.7	30.3 ± 2.4

Rab 7	15.2 ± 1.2	36.6 ± 2.0

The co-localization coefficients presented reflect the percentage of APP-GFP pixels co-localizing with Transferrin, Rab 5 or Rab 7 pixels, respectively, in cytoplasmic vesicles only or in total cell. Total number of pixels in the APP-GFP channel is taken as 100%. Values are mean ± SEM (n=10).

**Figure 2:**
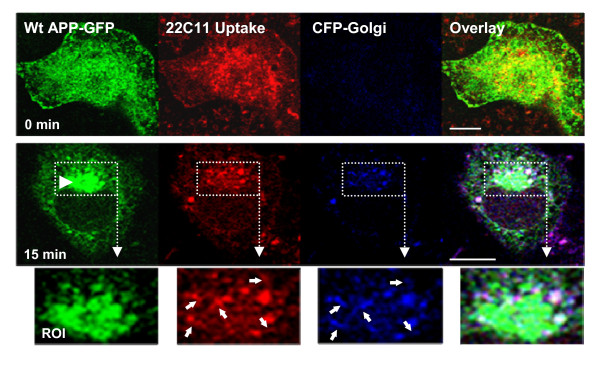
**A pool of endocytic APP-GFP is targeted to the TGN**. Wt APP-GFP at COS-7 cell surface was labelled by incubating cells with the 22C11 anti-APP ectodomain antibody ("22C11 Uptake", Texas red staining) at 4°C. At 0 min, 22C11 staining is maintained at the cell surface (microphotographs taken at the plasma membrane focal plane). Following 15 min at 37°C, part of the 22C11 population can be observed at vesicular structures around the Golgi (arrowhead; microphotographs taken at the Golgi focal plane). Endocytic 22C11/APP-GFP targeting to the TGN was confirmed by co-localization with a pECFP-Golgi construct ("CFP-Golgi", blue fluorescence), a construct targeted to the trans and medial region of the Golgi. Of note, since the CFP-Golgi fusion protein has a weak blue fluorescence, it is virtually non-visible at the plasma membrane focal plane. ROI - region of interest, 2.0-fold magnified. Several white vesicles, denoting 22C11/APP-GFP/CFP-Golgi co-localization, can be observed at the TGN (arrows in ROIs), A tubular morphology can be depicted for some of these vesicles in the ROIs. Bar, 10 μm.

The fate of endocytosed APP, in terms of retrograde retrieval, was subsequently analyzed making use of the APP-GFP construct and an antibody uptake assay. Wt APP-GFP expressing cells were incubated in ice-cold conditions in the presence of an antibody against the APP ectodomain (22C11). This antibody was raised against an APP ~50 aa N-terminal epitope, and recognizes only full length APP-GFP and not APP-GFP green fluorescent C-terminal peptides. Surface labeling of APP was achieved with 22C11 and endocytosis was monitored by tracking 22C11 uptake/APP-GFP co-localizing vesicles. Fig. 2, shows images taken at 0 min at 37°C, (cell surface focus plane), and subsequent images taken upon 15 min incubation at 37°C (focus plane through the Golgi), whereupon endocytosis had been allowed to proceed. By the end of the 15 min period, some of the co-localizing red 22C11/APP-GFP green vesicles, corresponding to full-length endocytosed APP-GFP, could be observed at the TGN area (Fig. 2, 15 min, white vesicles). The Golgi is the major subcellular site of APP enrichment [48], being easily identified as the intense juxtanuclear green fluorescent structure (Fig. 2, boxed area and [49]). Some of the 22C11 staining was confirmed to localize at the trans and/or medial Golgi regions, by co-transfection with an enhanced cyan fluorescence protein-Golgi (ECFP-Golgi) construct carrying a galactosyltransferase signal targeting it to the trans and medial-Golgi. Further morphologic analysis of these 22C11-positive vesicles at the TGN revealed tubular protuberances emerging from several of the vesicles, resembling the characteristic retromer-induced sorting tubules (Fig. 2, arrows in ROI). Of note, some of these tubular structures were also positive for the ECFP-Golgi construct, being co-transported with APP back to the TGN. This has been reported to occur in a retromer-dependent manner for several proteins e.g. TGN38/46 or furin that were used as TGN markers [30]. Thus it is clear that part of the endocytosed APP population is recycled to the TGN and sorted into tubular structures, suggesting a retromer-mediated pathway.

### APP is retrieved from endosome to TGN through clathrin and Rab5-positive vesicles

**Figure 3:**
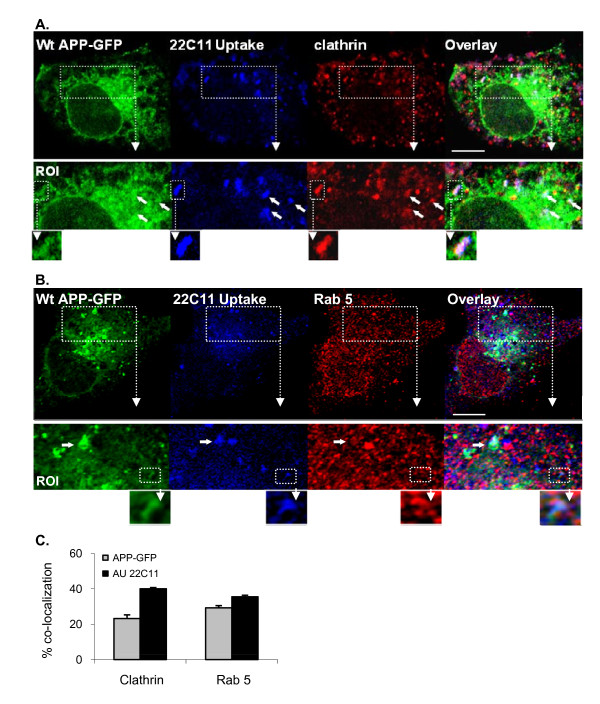
**Endocytosed APP is present in Clathrin and Rab 5-positive vesicles tubulating vesicles**. The 22C11 uptake assay (Alexa350 blue staining) was repeated in COS-7 cells for further characterization of the vesicles retrieving endocytosed APP-GFP to the TGN. Immunocytochemistry analysis using antibodies against **(A) **clathrin and **(B) **Rab 5 (Texas red staining) confirmed the presence of these proteins in APP-GFP endocytic vesicles. ROI, region of interest, 1.7- and 2.0-fold magnified, respectively. Arrows in ROIs depict APP tubulating endosomes, further magnified. While 22C11/APP-GFP and clathrin were observed to be sorted from the vacuole to the emerging tubule, Rab 5 appears to be maintained in the vacuole. Bar, 10 μm. **(C) **confocal quantitative analysis of the degree of co-localization between Wt APP-GPP and uptaken 22C11 with clathrin and Rab-5. Values are mean ± SEM, n = 20 cells.

Endocytosed APP-GFP vesicles were further analyzed for their co-localization with protein markers (Rab5 and clathrin) known to be present in intermediate endosomes destined for TGN retrieval via the retromer complex (Fig. 3). Surface internalized Wt APP-GFP (green APP-GFP/blue 22C11 co-localizing vesicles) could be observed to co-localize with Rab5 or clathrin (red staining) in whitish vesicles (white arrows in Fig. 3a and 3b ROIs, respectively). Co-localization quantitative analysis was performed (Fig. 3c), rendering the following results: 23.2 ± 2.1 and 29.2 ± 0.7% for Wt APP-GFP co-localization with Clathrin and Rab 5, respectively, increasing to 40.0 ± 1.3 and 35.5 ± 0,9% co-localization with uptaken 22C11 antibody for the same proteins. Moreover, the previously observed protruding tubules, emerging from the donor vesicle, could also be detected (Fig. 3a and b, depicted structure in ROIs). All our data are consistent with a retromer-mediated TGN retrieval for APP. To note, the definition at this photonic level is not very high, and higher resolution can only be obtained with electronic devices as has been observed by other authors [32,33]. Detailed evaluation reveals that the APP-GFP staining (green/blue co-localization) is present in the donor vesicle and in the emerging tubule (Fig. 3a and 3b, white depicted structure in ROI). The same appears to occur with the clathrin staining that, as far as can be observed, was also present in both parts of the structure (Fig. 3a, white depicted structure in ROI), in accordance with a potential role in assisting retromer in membrane curvature [33]. However, we could not observe the same for the Rab 5 staining. This appears to be only present at the donor pre-tubular vesicle (Fig. 3b, depicted structure in ROI), as reported for other early endosome proteins such as the transferrin receptor or EEA1 [33]. Further evidence for an APP retromer-mediated TGN retrieval was obtained by partially blocking retrograde transport to the TGN by incubating at 19.5°C [32,33]. Following 15 min at 19.5°C, co-localizing APP-GFP/endocytosed 22C11/clathrin white vesicles were found more distributed throughout the cytoplasm, there was obvious co-localization among the three stains and a marked decrease in the number of tubular structures detected (additional file 1). These changes are consistent with events associated with a temperature block as previously reported [32,33].

### PDBu enhances APP and VPS35 co-localization at the Golgi

The above results indicate that endocytosed APP is retrieved to the TGN via a trafficking route involving the retromer, of which VPS35 is a component. We have addressed the co-localization of these two proteins and alterations in response to PKC activation. In fact, APP and VPS35 co-localize to vesicular structures throughout the cell (additional file 2). Phorbol esters are known to alter APP processing [50], and interestingly, PKC activation resulted in altered APP/VPS35 distribution, increasing co-localization around the Golgi area (additional file 2). Protein kinase C (PKC) is known to phosphorylate APP only at S655, potentially enhancing the exit of APP-containing vesicles [44,50,51]. Mechanistically, we propose that APP phosphorylation within the sorting motif ^653^YTSI^656 ^may be involved both in protein sorting from and to the TGN, as seen for the sortilin, which has the YSVL sequence [42,43]. Hence, APP-GFP S655 phosphomutants were employed to determine the influence of S655 phosphorylation on APP endosomal traffic and related processing.

### Mimicking S655 phosphorylation results in enhanced mature APP half-life

**Figure 4:**
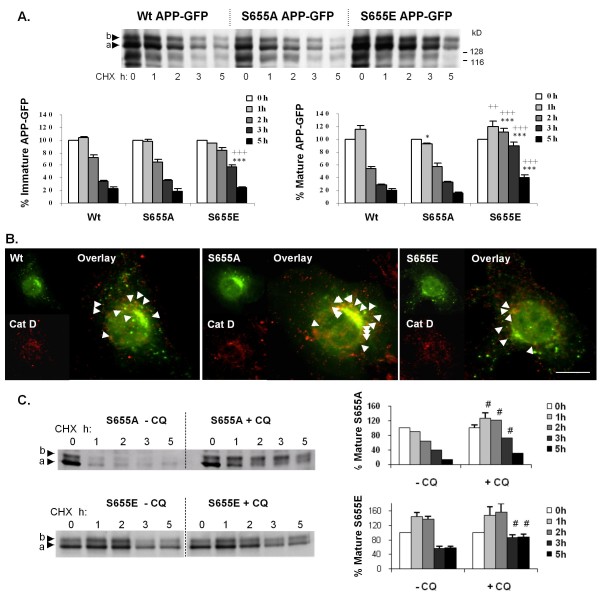
**S655 APP phosphomutants exhibit differential cellular catabolism**. **(A) **Wt and S655 phosphomutants turnover rates in COS-7 cells. Upper panel: Immunoblot analysis of APP-GFP transfected cells lysates using the anti-APP antibody 22C11. Bands a and b, immature (N-glycosylated) and mature (N- and O-glycosylated) APP-GFP forms, respectively, migrating above endogenous APP forms of ~115 kD and ~109 kD (potentially immature APP_751/770 _and APP_695_, respectively). APP-GFP bands identity was further confirmed using an anti-GFP antibody (data not shown). Lower panel: plotted data of the levels of immature (left graph) and mature (right graph) APP-GFP, expressed as percentage of OD values at 0 h in CHX. Values are mean ± SEM (n = 6). Statistical significance symbols: (*), S655 phosphomutants vs. Wt; (^+^), S655A vs. S655E; statistical significance levels are presented as (*/^+^) for *p *≤ 0.05; (^++^), for *p *< 0.01; and (***/^+++^) for *p *< 0.001. **(B) **S655A is preferentially delivered to lysosomes. The targeting of Wt and S655 phosphomutants (green vesicles) to lysosomes was analysed by co-localization with the lysosomal marker cathepsin D ("Cat D", red vesicles). Arrows indicate APP-GFP-containing lysosomes (green/red co-localizing vesicles). Bar, 10 μm. **(C) **Alterations in the mature S655A and S655E turnover upon inhibition of lysosomal hydrolases with 50 μM chloroquine (CQ). Left: Immunoblot analysis of transfected cells lysates using the anti-GFP antibody JL-8. Right: mature S655A and S655E (band b) levels were quantified and data plotted as percentages of OD values at 0 h in CHX. Values are mean ± SEM (n = 3); (^#^), statistical significance of *p *≤ 0.05 for CQ plus vs control data.

Cells were transfected with APP-GFP cDNAs carrying phosphomutations at S655 (dephosphomimetic S655A and phosphomimetic S655E) and the levels of APP-GFP proteins were monitored with time of CHX incubation. Immunoblotting analysis using the anti-APP 22C11 antibody (Fig. 4A) revealed two bands migrating at the expected molecular weights for the APP-GFP fusion proteins, band a (~136 kD) and band b (~145 kD), corresponding to the immature and mature APP-GFP species, respectively [46]. APP-GFP proteins matured similarly to endogenous APP [44], and APP-GFP maturation was unchanged by S655 phosphomutations; 35.0 ± 3.3% for Wt, 32.8 ± 2.7% for S655A and 34.0 ± 4.0% for S655E (calculated as the % of mature APP-GFP relative to total APP-GFP levels, at 0 h of CHX exposure). A representative profile of APP-GFP C-terminal peptides with CHX is presented in additional file 3. These peptides accounted for 25-50% of the GFP population at the 2h-3 h in CHX time interval.

The levels of the APP-GFP species with time in CHX were subsequently quantified and expressed as percentages of initial levels at time 0 h CHX (Fig. 4A). The rates of immature APP-GFP protein turnover were mainly found unaltered (Fig. 4A, left graph). All immature APP-GFP proteins (Wt, S655A, S655E) rapidly decreased with time in CHX and reached the same end point (~20% of initial levels at 5 h). Slight delays in immature S655E disappearance are most probably of no physiological significance, since immature APP is not normally phosphorylated at S655 [52]. In contrast, comparison of the mature APP-GFP time courses revealed differences in the turnover rates of the mature S655 mutants (Fig. 4A, right graph). Upon 5 h of CHX exposure, levels of Wt and S655A proteins decreased to 20% of initial levels, while levels of the S655E mutant only decreased to 40% (Fig. 4A, right graph). Additionally, the Wt had an initial positive slope (0-1 h) that was considerably augmented and sustained over time for the S655E mutant, but absent for S655A. The absence of this initial peak for S655A suggests that this form is more readily available for catabolism. In sharp contrast, the mature S655E levels decreased below 100% only after 2 and 3 h. Mature APP-GFP half-lives were subsequently calculated as 2.46 ± 0.16 h for the Wt, 2.12 ± 0.08 h for the S655A, and 5.56 ± 0.41 h (*p *< 0.001 vs Wt and S655A) for the S655E mutant. We further investigated whether S655-dependent rates of catabolism could be due to divergent sorting fates upon APP endocytosis, i.e., targeting to the TGN or to lysosomes.

### The dephosphomimetic S655A mutant is preferentially targeted for lysosomal degradation

Contrasting half-lives for the mature forms of S655 APP-GFP phosphomutants suggest differences in lysosomal degradation. The influence of S655 phosphorylation on APP lysosomal targeting was therefore analyzed by co-localization with the lysosomal marker cathepsin D (Cat D). APP-GFP expressing cells presented a typical subcellular distribution of Cat D (red staining) in small cytoplasmic vesicles, enriched near the Golgi area (Fig. 4B). The number of APP-GFP/Cat D co-localizing vesicles was low, ranging from 2 to 20 per cell, when compared with an average number of ~100 APP-GFP endocytic vesicles per cell (Fig. 1). These values are not surprising, given that APP is primarily detected in lysosomes when lysosomal proteolysis is inhibited [49,53]. Transfected cells were thus monitored for the extent of APP-GFP/Cat D co-localization (Table 2). The percentage of green/red co-localizing vesicles relative to total APP-GFP green cytoplasmic vesicles was scored (Table 2, "Vesicular co-loc."), and microphotographs were analyzed by a Zeiss confocal software to determine APP-GFP/Cat D co-localization coefficients (Table 2, "Co-loc. coefficient"). Both approaches rendered similar values and confirmed the visual observation that lysosomal targeting was diminished in the S655E mutant, whereas in the S655A mutant APP-GFP co-localization with CatD was enhanced. Consequently, cells expressing S655A and S655E were exposed to chloroquine (CQ), a drug that neutralizes lysosomal pH, thereby partially inhibiting mature APP lysosomal degradation [49]. CQ was able to shift the basal pattern of mature S655A catabolism to resemble a S655E-like pattern (compare Fig. 4C graphs), while the mature S655E profile only suffered slight increases upon CQ treatment. This clearly associates S655 phosphorylation state with APP-GFP catabolism via lysosomal degradation.

**Table 2:** Co-localization of the S655 phosphomutants with the lysosomal marker cathepsin D.

	Parameters of APP-GFP/Cat D co-localization	
**APP-GFP**	**% Vesicular co-loc**.	**% Co-loc. coefficient**

Wt	5.0 ± 0.5	6.4 ± 0.7

S655A	9.6 ± 0.4***^/+++^	10.2 ± 0.8***^/+++^

S655E	3.1 ± 0.3^+++^	3.6 ± 0.0*^/+++^

The number of APP-GFP/cathepsin D co-localizing vesicles was scored and expressed as percentage of APP-GFP-containing vesicles ("Vesicular co-loc."). The co-localization coefficients ("Co-loc coefficient") represent the relative percentage between the number of APP-GFP/cathepsin D co-localizing pixels and the total number of pixels of the APP-GFP channel, upon single cell delimitation. Values are mean ± SEM (n = 20). (*), S655 mutant vs Wt data; (^+^), S655A vs S655E data. *, p < 0.05; ***/^+++^, p < 0.001.

### S655 phosphorylation is a targeting signal for APP retrieval to the TGN

**Figure 5:**
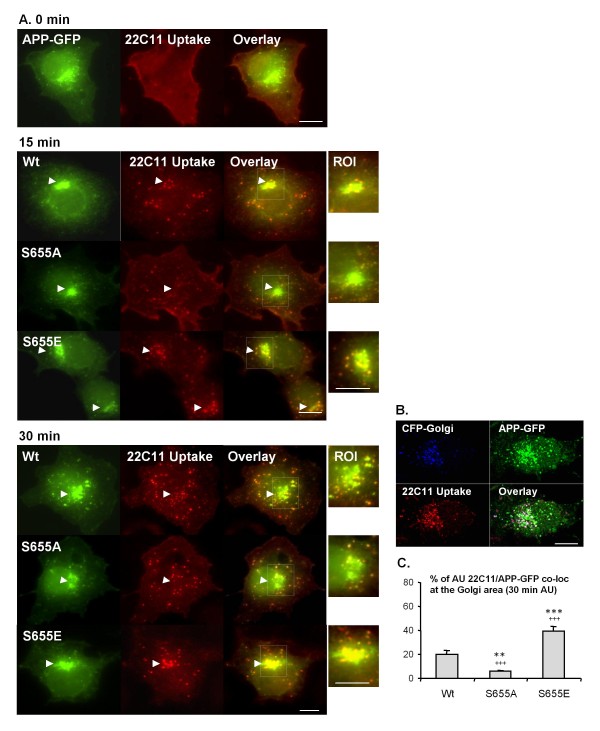
**S655 phosphorylation is a targeting signal for retrieval of APP to the Golgi**. **(A) **Wt, S655A and S655E APP-GFP at the cell surface were labelled (0 min) by incubating COS-7 cells with the 22C11 anti-APP ectodomain antibody ("22C11 Uptake", Texas red staining) at 4°C. Following 15 and 30 min of incubation at 37°C, the localization of APP-GFP endocytic vesicles (green/red-22C11 co-localizing vesicles) were monitored. Arrows indicate the Golgi area and APP-GFP/22C11 endocytic vesicles co-localizing to or around the same region. ROI - region of interest denoting the Golgi area and co-localizing vesicles. **(B) **The highly green-fluorescent perinuclear structure in APP-GFP transfected cells was confirmed to be the Golgi in Wt APP-GFP and CFP-Golgi co-transfected cells, upon 30 min of 22C11 uptake. Bar, 10 μm. **(C) **confocal quantitative analysis of the degree of co-localization between uptaken 22C11 and the APP-GPP population at the Golgi area at 30 min of 22C11 antibody uptake (AU) at 37°C. Values are mean ± SEM, n = 20 cells. Statistical significance symbols used were: (*) for comparison of S655 phosphomutant and Wt data; (^+^) S655A vs S655E data. Statistical significance levels are presented as (**), for *p *< 0.01; and (***/^+++^), for *p *< 0.001.

The half-life of mature S655E was doubled, consistent with it being sorted away from lysosomal degradation. Enhanced S655E TGN retrieval supports its deviation from the default lysosomal pathway, resulting in increased recycling. Therefore the antibody uptake assay (Fig. 2 and 3) was repeated for all APP-GFP constructs (Wt, S655A and S655E) and the dynamics of endocytic APP retrieval to the TGN were monitored with time at 37°C (Fig. 5). Epifluorescence microscopy was first used for an overview of all the APP-GFP endocytic vesicles, and confocal microscopy was used for quantification analysis of targeting to the Golgi area.

As previously observed (Fig. 2), at 0 min, a strong 22C11 antibody red staining co-localizing to all APP-GFP proteins could be observed at the cell surface (Fig. 5a, 0 min). Upon 15 min of endocytosis, differences could be detected in the location of the APP-GFP positive endocytic vesicles, depending on the construct being expressed. A semi-quantitative approach was used to study retromer-mediated recycling [54]. At 15 min at 37°C, some of the endocytosed Wt and S655E APP-GFP vesicles were found near or at the Golgi area, but more so for the S655E protein (Fig. 5a, 15 min). Of note, the main fluorescent perinuclear structure in APP-GFP expressing cells was confirmed as the Golgi area using the ECFP-Golgi construct, in APP-GFP/ECFP-Golgi co-transfected cells upon 30 min 22C11 uptake (Fig. 5b). This distribution occurred in 50% of S655E-expressing cells, but only in 30% of Wt-expressing cells. In contrast, co-localization of endocytic S655A vesicles at the Golgi area was largely undetectable (only visible in 7% of S655A-expressing cells), and mainly remained randomly distributed throughout the cells' cytoplasm (Fig. 5a, 15 min). By 30 min at 37°C, 22C11/APP-GFP endocytic vesicles co-localizing at the Golgi area increased to 70% of S655E-expressing cells, compared to 50% for Wt and 40% for S655A. Furthermore, percentage co-localization of endocytosed APP (22C11 Uptake) with the APP-GFP population at the Golgi area was determined for each of the three proteins using the Zeiss confocal software (Fig. 5c). Results for the three APP-GFP proteins supported previous observations, namely that the phosphomimicking S655E undergoes enhanced recycling: 20.3 ± 2.9% for Wt, 5.7 ± 0.8% for S655A, and 39.2 ± 3.9% for S655E (n = 20 cells; p < 0.001 for S655E data vs Wt or S655A data; p < 0.01 for Wt vs S655A data).

In order to confirm that we were monitoring endocytosed APP, cell permeabilization was omitted in some experiments and, in these conditions, vesicles typical of endocytosis (as in Fig. 3 and 5, for example) were not visible, but rather diffuse dot-like staining could be visualized at the plasma membrane (additional file 4).

### S655 phosphorylation dependent APP retrieval to the TGN is potentially mediated by the retromer complex

**Figure 6:**
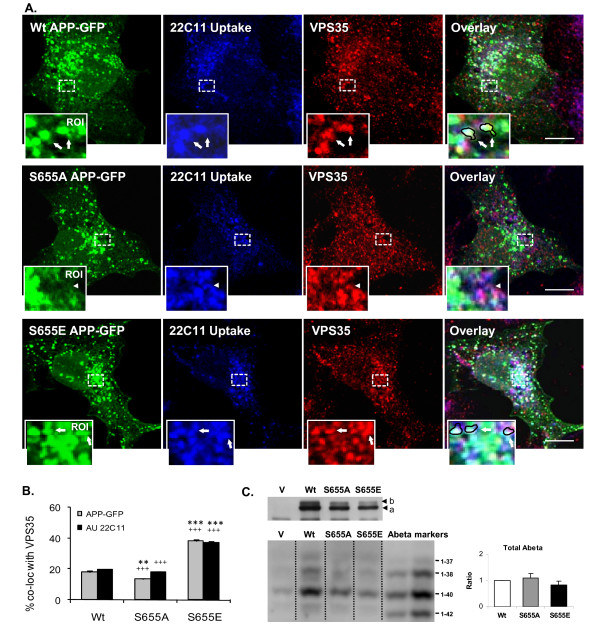
**S655 phosphorylation-dependent APP retrieval occurs via VPS35-containing endocytic vesicles**. **(A) **APP-GFP transfected COS-7 cells were subjected to the 15 min 22C11 uptake assay (Alexa350 blue staining) and the co-localization of endocytosed APP-GFP proteins (blue/green vesicles) with the VPS35 retromer subunit (Texas red staining) was monitored. The Golgi area denotes the highest degree of co-localization, and a region was magnified for better visualization of the tubulating vesicles (ROI-4.0-fold magnified; arrows point to APP-GFP/AU22C11/VPS35-positive structures for Wt and S655E, and arrowhead to a S655A APP-GFP-negative, AU22C11/VPS35-positive structure). Some of the tubulating vesicles for Wt and S655E have been represented schematically with a black line. **(B) **Co-localization between APP-GFP or 22C11 uptake with VPS35 was quantified using confocal software. Values are mean ± SEM, n = 30 cells. Statistical significance symbols: (*), S655 phosphomutant vs Wt data; (^+^), S655A vs S655E data. Statistically significant levels are presented as (**) for *p *< 0.01; and (***/^+++^) for *p *< 0.001. **(C) **Secreted Abeta peptides from 3 h conditioned media of APP-GFP or EGFP vector ("V") transfected COS-7 cells. Samples were immunoprecipitated, separated on urea gels and immunostained with mAb 1E8. Upper panel: Immunoblot analysis of cells lysates using the anti-APP antibody 22C11. Bands a and b, transfected immature and mature APP-GFP forms, respectively. The various Abeta species were quantified, corrected for the relative respective APP-GFP expression levels (bands a+b), and plotted as fold-increase of total (sum of all species) Abeta fragments over Wt values (mean ± SEM, n = 3).

All the above data strongly suggest that TGN retrieval of APP is mediated by the retromer complex in a S655-phosphorylation state dependent manner. Further data strengthening this hypothesis arise from co-localization studies of endocytosed Wt, S655A and S655E with the retromer protein component VPS35. The latter occurs endogenously in COS-7 cells [55]. The previous 22C11 antibody uptake assay was used, and the degree of co-localization between endocytosed APP and VPS35, at a focal plane crossing the Golgi apparatus, was determined using the Zeiss confocal software. Microphotographs (Fig. 6a) show that all three APP-GFP proteins endocytosed co-localize with VPS35-containing vesicles. For each APP-GFP protein, endocytosis was confirmed given the co-localization of the blue 22C11 fluorescence and green APP-GFP fluorescence. Co-localization with the VPS35 derived red staining was evident throughout the cytoplasm, but to a higher extent near or at the Golgi area (Fig.6a). This is clearly indicative of APP-GFP endosomal-to-TGN targeting being mediated by the retromer complex, and consistent with all the above data. Notably, some of the vesicular structures where the three stainings co-localize (white structures, circumference drawn by a black outline in the ROI, Fig.6) have the "tubular budding" morphology characteristic of retromer-mediated membrane deformation (see Fig. 6a, arrows in ROI). APP delivery to the TGN appears to occur through these tubular structures upon the TGN targeting of the APP-containing intermediate endosomes. Further, and of central importance, a higher co-localization between endocytosed S655E APP-GFP and VPS35 could be easily observed, in particular when compared to the S655A mutant that exhibited the lowest degree of co-localization (Fig. 6a, arrowhead in ROI). The co-localization coefficients of the APP-GFP population with VPS35 and the uptaken 22C11 with VPS35 were evaluated for each APP-GFP protein (Fig. 6b). The results revealed a marked difference for the S655E mutant. Wt APP-GFP co-localized with VPS35 by 18.0 ± 0.9%, the S655A mutant by 13.4 ± 0.4%, while co-localization values for the S655E doubled to 38.0 ± 1.2% (Fig 6b, grey columns). The values obtained for the 22C11 uptake and co-localization with VPS35 were similar (Fig. 6b, black columns), confirming that endocytosed full-length APP-GFP proteins were being monitored.

Together, the data prove a S655 phosphorylation-enhanced APP TGN retrieval, and strongly suggest this to be retromer-mediated. The TGN retrieval pathway, involving SorLA and the retromer, has been inversely correlated with Abeta production. Therefore, the levels of Abeta were also analyzed in the 3 h conditioned media of transfected cells. For each APP-GFP protein, all the individual Abeta species detected followed similar fold-increases. Their sum is here presented graphically (Fig. 6c, lower blot, 'total Abeta'), upon correction for holo APP-GFP relative transfection levels (ratio between APP-GFP levels, each calculated by the sum of bands a+b in Fig. 6c, upper blot). The differences between Abeta amounts produced by the APP-GFP proteins under CHX revealed some differences. However, levels were very low and difficult to measure reliably (data not shown). Thus, comparative Abeta production was measured in 3 h CHX-free media, resulting in less marked differences due to continuous expression of APP-GFP. Nonetheless, a tendency towards lower total Abeta production for S655E (0.83 ± 0.13 of Wt values) is observed, consistent with a lower time of residence in the endosomes for this mutant, due at least in part, to faster recycling back to the TGN.

### APP co-immunoprecipitates with VPS35 and SorLA

**Figure 7:**
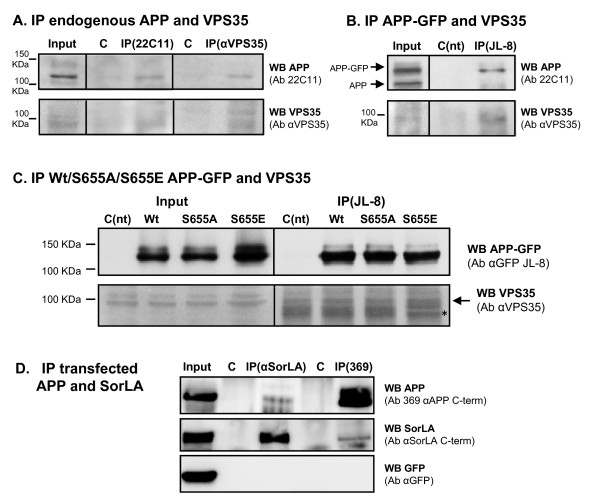
**Co-immunoprecipitation of APP, VPS35 and SorLA in COS-7 and HEK293 cells**. **(A) **Endogenous APP and VPS35 were co-immunoprecipitated in COS-7 cells using the anti-APP N-terminus 22C11 and the anti-VPS35 antibodies (αVPS35). Negative controls (C) were performed by immunoprecipitating cells with the same secondary antibodies, sepharose- (IP VPS35 control) and agarose- (IP 22C11) linked, respectively. **(B) **Transfected Wt APP-GFP and endogenous VPS35 co-immunoprecipitate from COS-7 cells using the indicated antibodies (Ab). **(C) **Transfected Wt, S655A and S655E APP-GFP were immunoprecipitated in COS-7 cells with the anti-GFP antibody, and the co-immunoprecipitated endogenous VPS35 forms were detected with an anti-VPS35 antibody. Asterisk (*), non-specific IgGs bands (IgGs raised in goat) of the IP samples. Non-transfected cells (C(nt)) submitted to the same IP procedures (incubated with primary and agarose-linked secondary antibodies) were used as control in **(B) **and **(C)**. **(D) **HEK293T cells were cotransfected with SorLA cDNA, APP_695 _and eGFP (transfection control). Immunoprecipitation was performed using antibodies raised against the C-terminus of APP (369), the N-terminus of SorLA (αSorLA) or using pre-immune serum as negative controls (C). Immunoprecipitation and co-immunoprecipitation were detected by western blot (WB) using the anti-APP 369 antibody and an anti-SorLA C-terminus antibody (αSorLA C-term). Immunoblot analysis included GFP as an additional negative control.

To analyze the formation of APP/VPS35 and APP/SorLA complexes, immunoprecipitation assays were further conducted. APP, whether endogenous or expressed as APP-GFP, was found to co-immunoprecipitate with endogenous ~95kDa VPS35 (Fig. 7a,b,c). Although the endogenous signal for VPS35 is weak, it is clear that APP and VPS35 co-immunoprecipitate (Fig. 7a,b). For comparison purposes, the IP was repeated for the three Wt/S655A/S655E APP-GFP proteins, and a higher level of VPS35 was observed to co-IP with S655E (Fig. 7c), suggesting that S655 phosphorylation enhances APP binding to the retromer complex. Further, APP-VPS35 binding may be direct or through SorLA as a bridging protein. Indeed, when co-expressed in HEK293T cells, mature APP_695 _co-immunoprecipitated with SorLA and mature SorLA co-immunoprecipitated with APP_695 _(Fig. 7D). These results agree with previous studies by [56,57] showing an interaction between APP and SorLA. Of note, APP and SorLA C-terminal fragments (CTF) did not co-immunoprecipitate, and the additional negative control, eGFP, also failed to co-immunoprecipitate with either SorLA or APP.

### Downregulation of VPS35 impairs endocytosed APP retrieval to the TGN

**Figure 8:**
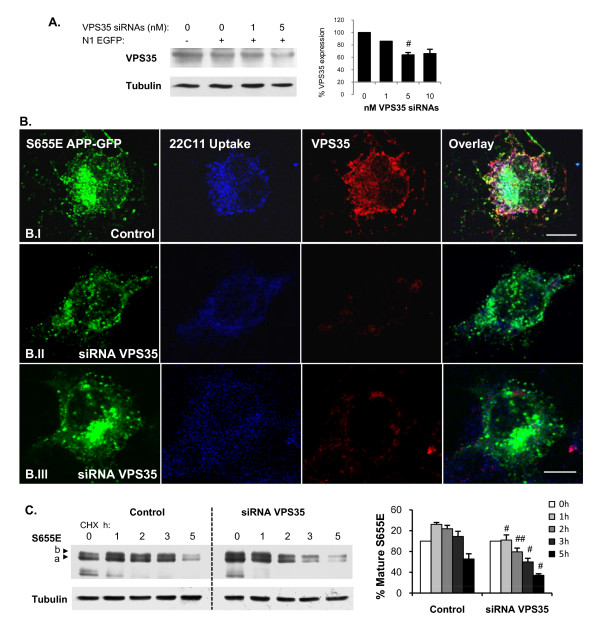
**VPS35 down-regulation impairs S655E APP-GFP retrieval to the TGN and decreases its turnover rate**. **(A) **Left: Immunodetection of VPS35 levels in COS-7 cells co-transfected with N1 EGFP and several concentrations of anti-VPS35 siRNAs for 24 h. Tubulin was probed as a control. Right: Quantification of VPS35 levels, plotted as percentages of control conditions (cells only transfected with N1 EGFP). Values are mean ± SEM (n = 2-6); (#), statistical significance of p ≤ 0.05 for siRNA VPS35 vs control data. **(B) **The 15 min 22C11 antibody uptake assay (Alexa350 blue staining) was applied to monitor S655E APP-GFP protein retrieval from the endosome to the TGN upon COS-7 cells pre-incubation with 5nM VPS35 siRNAs for 24 h to downregulate VPS35 (Texas red staining). **(B.I) **Control: cells only transfected with S655E APP-GFP. **(B.II - B.III) **siRNA VPS35: cells co-transfected with S655E APP-GFP and VPS35 siRNAs; in the majority of the population, the APP-GFP signal at the Golgi was not visible **(B.II**), while in a minor percentage of cells APP-GFP was still visible at the Golgi **(B.III)**. Bar, 10 μm. **(C) **S655E APP-GFP turnover rate in CHX upon cells pre-incubation with 5nM VPS35 siRNAs for 24 h. Left: Immunoblot analysis of COS-7 transfected cells lysates using the anti-APP 22C11 and anti-tubulin antibodies. Bands a and b, immature and mature APP-GFP forms, respectively, migrating above endogenous APP forms. Right: mature S655E levels were quantified and plotted as percentages of OD values at 0 h in CHX. Values are mean ± SEM (n = 3); (#), statistical significance of p ≤ 0.05 for siRNA VPS35 plus vs control data.

Given the central role proposed for the retromer complex, it became essential to analyze the effects of VPS35 down-regulation on S655E APP-GFP retrieval to the TGN and on this mutant's half-life. VPS35 expression was decreased by means of siRNA transient transfection. VPS35 down-regulation conditions were first optimized, with the levels of VPS35 expression being the lowest (64 ± 4%, n = 6, p < 0.05 vs control data) for 5 nM of VPS35 siRNAs transfection upon 24 h of expression, while 10 nM resulted in a similar decrease (Fig. 8A). Of note, transfection of 5 or 10 nM of VPS35 siRNAs resulted in a decrease of sAPP secretion into the medium to ~80% of control levels (data not shown).

The 22C11 antibody uptake assay was subsequently repeated in COS-7 cells transiently co-transfected with the S655E APP-GFP cDNA and 5 nM of the VPS35 siRNAs for 24 h, followed by 2h30 h in CHX (Fig. 8B). A clear decrease in the S655E APP-GFP signal was visible in cells where VPS35 expression (red labeling) was down-regulated (Fig. 8B.II siRNA VPS35). Further, the APP-GFP signal was found in cytoplasmic vesicles and was absent from the Golgi of the majority of these cells (65.0 ± 8.0% of the population). This is in contrast with what occurs in control cells (Fig. 8B.I), where the S655E APP-GFP signal was clearly visible at the Golgi of 83.0 ± 3.7% of the cells (p < 0.01, n = 3 independent experiments where 30-100 cells were scored). Low levels of endocytosed APP (AU 22C11, blue staining) were observed in cells where VPS35 was down-regulated, most probably resulting from the observed decrease in APP-GFP levels. Most importantly, in ~90% of the siRNA VPS35 transfected cells, the endocytosed 22C11 vesicles were found diffusely distributed throughout the cytoplasm (Fig. 8B, 22C11 uptake), in contrast with the normal localization around and at the TGN in control cells (Fig. 8B.I, 22C11 uptake). Noticeably, similar 22C11 vesicular distribution was also observed in the smaller percentage of the population (35.0 ± 8.0%) where APP-GFP was still visible at the Golgi (Fig. 8B.III). Thus, down-regulation of cellular VPS35 levels resulted in less S655E APP-GFP at the Golgi and impaired APP retrieval from the endosome to the TGN.

The effect of VPS35 down-regulation on the half-life of the S655E APP-GFP mature form was also addressed (Fig. 8C), using the approach discussed above. Strikingly, cell pre-incubation with VPS35 siRNAs for 24 h resulted in both an increase in APP-GFP levels (0 h in CHX) and in an increase in the APP-GFP and endogenous APP turnover rates. Indeed, VPS35 down-regulation induced a shift in the basal pattern of mature S655E catabolism towards a more S655A-like pattern (compare Fig. 8C and 4A graphs). Indeed, the typical initial (0-1 h) positive slope in the S655E APP-GFP profile is completely abolished when VPS35 is downregulated, and mature S655E APP-GFP half-life decreased 2.1 ± 0.3 fold. Further, the diminished levels of APP-GFP at 2-3 h CHX are in agreement with the observed lower APP-GFP and 22C11 uptake signals observed in Fig. 8B. These results conclusively associate the retromer complex with APP-GFP catabolism and its retrieval to the TGN upon its endocytosis.

## Discussion

The time of residence associated with APP traversing the endosomal pathway is critical to its processing and appears to correlate with Abeta levels and AD pathogenesis [28,58,59]. Endosomes are known sorting stations, crucial to understanding AD [21-24,37], but the molecular mechanisms underlying endosomal APP sorting and trafficking are not clearly defined [4,28]. In the work here described, we observed that endocytosed APP molecules can be sorted for rapid retrieval to the TGN, in a retromer-mediated manner. Although we have found vesicle tubulation outside the TGN vicinity, our results suggest that tubulation occurs to a higher extent in APP-containing intermediate endosomes near the TGN (Fig. 2). Hence, the nascent tubule appears to be directly responsible for the delivery of retrieved APP cargo to the TGN. As previously reported, at the protein level, the intermediate endosomes are positive for clathrin and for the early endocytic marker Rab5, although with apparent minor differences in their distribution (Fig. 3). We have also shown that, at least at the photonic level of resolution used, Rab5 appears not to be sorted to the emerging tubule, while clathrin was present in this nascent structure (ROIs in Fig. 3). Other early endosome markers, such as EEA1 [33], present a distribution similar to that observed by us for Rab5 in the intermediate endosomes destined for the TGN. The co-localization of APP with a retromer component related to cargo recognition, VPS35, strongly suggested it as a retromer-mediated pathway.

Since components of the retromer-mediated pathway and endocytic APP fate have been associated with AD pathology, we found it particularly important to address the regulatory signals determining retrieval of APP to the TGN. Transmembrane protein trafficking in the post-TGN membrane system may contain several sorting signals regulating protein transport between the various compartments [41]. For example, CIMPR undergoes retromer-dependent retrieval to the TGN and its cytoplasmic tail has both an YXXϕ and a DXXLL motif. These are motifs known to be involved in retromer-dependent BACE-1 and sortilin retrieval to the TGN, respectively [41,42]. APP also has a characteristic YXXϕ sorting signal, ^653^YTSI^656^, well positioned in the juxtamembrane region of the cytoplasmic tail [60] that could support both sorting at the TGN and TGN retrieval, as recently observed for the YXXϕ motif in sortilin [42]. The ^653^YTSI^656 ^functional motif was first related to APP endocytosis and post-TGN degradation [61-66], and lately to AP-1-binding dependent APP basolateral sorting in epithelial cells [43]. Further, protein cargo phosphorylation near or at the sorting motif could be a positive modulator for its retrieval to the TGN. Indeed, this occurs with CIMPR and BACE phosphorylation at serine residues near their cytoplasmic sorting DXXLL motif [41,67-69].

Protein phosphorylation is a major regulatory process, and APP phosphorylation is known to alter its subcellular processing [16,46,60,64,65,70,71]. Although phosphoS655 APP molecules, within the APP ^653^YTSI^656 ^motif, have been reported in AD brains [65,72,73], a clear physiological role for S655 phosphorylation, first proposed to regulate APP sorting by Gandy et al. [51], has not been forthcoming. We have recently observed that APP phosphorylation at S655 enhances the protein exit from the TGN to the PM and increased its cleavage to αsAPP [44]. Together with the data described here, one can conclude that S655 phosphorylation is important in regulating APP traffic from the TGN to the PM and in recycling APP back to the TGN. In fact, as we demonstrated, S655 phosphorylation has a key modulatory role in the sorting fate of endocytosed APP molecules. The phosphomimetic APP^S655E^, undergoes faster and enhanced retrieval to the TGN (Fig. 5). Further characterization of S655-dependent sorting at endosomes revealed that endocytosed APP^S655A ^was preferentially targeted to the lysosomal default route (Fig. 4). Similarly, retromer impairment has also been observed to promote Sortilin and the Shiga toxin B-subunit targeting to the lysosomal pathway [33,42]. The differential sorting of the APP mutants at endosomes, for TGN retrieval or lysosomal delivery, were reflected in the half-lives of their APP-GFP mature forms (Fig. 4). This confirmed a correlation between S655 phospho-state dependent endosomal sorting and APP-GFP turnover rates. The validation that S655 phosphorylation dependent APP retrieval to the TGN occurred in a retromer-mediated manner is confirmed by the VPS35 siRNA downregulation assays, where APP retrieval to the TGN and APP half-life were significantly reduced (Fig. 8). Importantly, we have observed a tendency for less Abeta production for APP^S655E ^that may be a result of its shorter time of residence in endosomes due to more rapid retrieval to the TGN. This agrees with reports inversely correlating components of this pathway with Abeta production and AD [21,36-38,59,74].

From a molecular mechanistic perspective, APP S655 phosphorylation appears to lead to an increase in its binding to sorting proteins, as occurs with the phosphorylation of BACE-1 and CI-MRP, wich enhance their binding to GGA, a protein involved in this transport [41,67-69,75,76]. In agreement with this, NMR analysis of S655 phosphorylated APP was found to induce significant local conformational changes in the APP C-terminus at and downstream the ^653^YTSI^656 ^motif [77]. Accordingly, we have observed more VPS35 immunoreactivity when VPS35 was co-immunoprecipitated with the S655E mutant (Fig. 7c). Nonetheless, Wt and S655A co-immunoprecipitated with VPS35 to similar extents, suggesting that increased S655A targeting to lysosomes involves not a default passive but a mediated active process, involving lysosomal sorting molecules. Other reports have indicated that retromer binding to cargo proteins most likely occurs via its VPS10-containing sorting receptor proteins, such as SorLA, and not via direct binding of cargo to VPS35 [34]. Noticeably, SorLA can bind GGA [39], and both can bind the clathrin AP-1/2 adaptor proteins [27], and all are reported to play roles in retrograde retrieval of cargo [21,27,75]. In light of our results, we speculate that S655 phosphorylation enhances APP binding affinity for sorting proteins such as SorLA [57] and/or the AP-1 adaptor, which function to retrieve APP to the TGN in a complex containing SorLA, AP-1, GGAs and the retromer.

## Conclusions

For the first time we show that APP is retrieved from the endosome to the TGN in a retromer-mediated pathway, and that this process is positively regulated by APP phosphorylation at its S655 residue. We have proved that the phosphorylation state of S655 is determinant in endocytic APP sorting to the TGN or lysosomes. S655 lies within the basolateral sorting APP motif, an important motif for APP binding to targeting proteins, such as SorLA and the retromer-related VPS35 protein. APP phosphorylation-dependent targeting is highly relevant from an AD therapeutic perspective. The pathogenesis of sporadic AD may be caused, at least in part, by impaired protein retrieval to the TGN [21,22,24,36,37]. Impairments of various cellular phosphorylation systems have been widely reported in AD [78-80], and may likewise be relevant to the disease condition. Retrieval of APP and BACE-1 to the TGN occur in a retromer- and phosphorylation-dependent manner, and a failure in one or both of these mechanisms would be predicted to result in higher endosomal co-compartmentalization of both proteins and enhanced amounts of generated Abeta. Therefore, the retromer-mediated process and its regulation by phosphorylation state of its cargo are both potential pathogenic factors underlying AD, and possible targets for future therapeutic strategies.

As further evidence for the key role of VPS10 domain proteins in AD, Andersen et al [81] and Lane et al [82] have recently demonstrated that SorLA modulation of APP metabolism requires binding of the SorLA cytoplasmic tail to VPS35 [81], and that another VPS10-domain protein, SorCS1, modulates coordinate risk of Alzheimer's disease and type 2 diabetes, apparently by controlling levels of both SorLA and VPS35 [82]

## Materials and methods

### Antibodies

Primary monoclonal antibodies used were 22C11 (Chemicon) against the APP ectodomain, JL-8 (BD Biosciences) for detection of the GFP moiety in APP-GFP proteins, the 1E8 monoclonal antibody (Nanotools, Germany) for Abeta detection, and two anti-APP C-terminal antibodies (rabbit anti-APP C-terminal, Zymed; rabbit APP C-terminal 369 antibody). Co-localization studies were carried out with anti-Rab5 (early-endosomal marker) (StressGen Bioreagents) and anti-Rab7 (late-endosomal marker) (CytoSignal) polyclonal rabbit antibodies, polyclonal goat clathrin antibody (ICN Immunobiologicals), anti-cathepsin D (lysosomal marker) monoclonal antibody (BD Biosciences), and polyclonal anti-VPS35 C-20 goat antibody (Santa Cruz Biotechnology). Immunoprecitipation and detection of SorLA was carried out using anti-N-terminal SorLA (BD transduction labs) or anti-SorLA C-terminal antibody raised by Dr. James Lah; an anti-GFP antibody (Sigma) was used in the IP controls. Secondary antibodies used were Texas Red-conjugated IgGs, Alexa Fluor 350-conjugated anti-rabbit IgGs, Alexa Fluor 568-conjugated anti-goat IgGs (Molecular Probes) and FITC-conjugated anti-rabbit IgGs (Calbiochem) for immunocytochemistry analyses, and horseradish peroxidase-linked IgGs antibodies (GE Healthcare) for enhanced chemiluminescence (ECL) detection.

### Wt and S655 Phosphomutants APP-GFP cDNAs

APP isoform 695 (APP_695_) cDNA was used as template to generate S655 cDNA point mutations, namely Serine 655 to Alanine (S655A) or to Glutamate (S655E), using site-directed mutagenesis [83]. These two amino acids, due to their size and charge, mimic a constitutively dephosphorylated and phosphorylated S655 residue, respectively. To engineer the APP_695_-GFP cDNA constructs (APP-GFP), the stop codons of Wt and S655 phosphomutants APP_695 _cDNAs were removed by PCR using specifically designed primers. The resultant fragments were digested with endonucleases (*Age*I and *Nru*I) and subcloned into the *Age*I/*Sma*I restriction sites of the GFP-encoding mammalian expression vector (pEGFP-N1, Clontech) as N-terminal APP-GFP translational fusions. The nucleotide sequences of the APP_695 _phosphorylation cDNA point mutants and the open reading frames were confirmed by DNA sequencing (ABI PRISM 310 genetic Analyser, Applied Biosystems).

### Co-localization of APP-GFP with endosomal markers

The endocytic pathway of the Wt APP-GFP protein was first assayed using Texas-red conjugated transferrin molecules (Molecular Probes) [84]. Monkey kidney COS-7 cells were maintained with Dulbecco's modified Eagle's medium (DMEM, Sigma) supplemented with 10% (v/v) fetal bovine serum (FBS, Gibco), 100 U/ml penicillin/100 mg/ml streptomycin (p/s) and 3.7 g/l NaHCO_3 _(complete DMEM) at 37°C and 5% CO_2_. COS-7 cells were grown on 35 mm plates containing pre-treated coverslips, with antibiotic/antimycotic (p/s)-free DMEM until 90% confluent, and transiently transfected with low levels of the APP-GFP cDNAs for 12 h. Transfections were performed using the cationic lipid LipofectAMINE 2000 (Invitrogen Life technologies), according to the supplier's instructions. Transfected cells were further exposed for 2:15 h to 50 μg/ml of the protein synthesis inhibitor cycloheximide (CHX, Sigma), in p/s-free DMEM. The experimental conditions for CHX drug dose and time of exposure were previously optimized [45,46]. Upon three washes with DMEM, cells were subsequently incubated for 30 min at 37°C with p/s- and FBS-free DMEM supplemented with 20 mM HEPES and 50 μg/ml CHX, to deplete endogenous transferrin. Medium was replaced with medium containing 1 mg/ml BSA and 100 nM Texas red-conjugated transferrin, and cells incubated for a further 15 min at 37°C. The plates were immediately cooled to 4°C and washed twice with ice-cold PBS. Cells were methanol-permeabilized and fixed with a 4% paraformaldehyde PBS solution. Additionally, two sets of Wt APP-GFP transfected cells were incubated for 3 h with 50 μg/ml CHX, fixed and submitted to immunocytochemistry procedures using the anti-Rab5 and anti-Rab7 antibodies diluted in a 3% BSA PBS solution. Coverslips were mounted on microscope slides with Fluoroguard Antifading Reagent (Bio-Rad) and analyzed by epifluorescence microscopy and by confocal microscopy (quantitative analysis).

### APP-GFP antibody uptake assays

COS-7 cells grown on polyornithine-coated glass coverslips were transiently transfected for 12 h with low levels of Wt or S655 phosphomutant APP-GFP cDNAs, as described above, and incubated with 50 μg/ml CHX for 2:30 h. An antibody uptake (AU) assay previously used to characterize BACE-1 phosphorylation-dependent TGN retrieval (Walter et al., 2001) was adapted. Briefly, cells were washed twice with ice-cold PBS and incubated for 20 min on ice, in FBS-free DMEM containing the 22C11 (anti-APP ectodomain) antibody. Upon three washes with ice-cold PBS, cells were subsequently incubated at 37°C in FBS-plus DMEM (10% FBS) for the indicated time points (0, 15, or 30 min). At each time point and after two washes with PBS and permeabilization with methanol, cells were fixed in 4% paraformaldehyde, and processed for immunocytochemistry with the antibodies indicated. To confirm endocytic Wt APP-GFP targeting to the TGN/Golgi, cells were co-transfected with a pECFP-Golgi construct (pEnhanced Cyan Fluorecent Protein-Golgi, Clontech), which encodes a fusion construct of ECFP and a sequence of the human beta 1,4-GT (galactosyltransferase), targeting it to the trans and medial region of the Golgi apparatus. 22C11 endocytic vesicles were visualized using an anti-mouse Texas red-conjugated antibody. In the co-localization assays, 22C11 endocytic vesicles were visualized using Alexa Fluor 350-conjugated antibody, and Rab5 or clathrin were detected using an Alexa Fluor 568-conjugated antibody. For the retromer co-localization assay, the endogenous VPS35 monomer was detected using an Alexa Fluor 568-conjugated anti-goat antibody.

### Immature and mature holo APP-GFP turnover rates

For a time-course analysis of APP-GFP turnover, COS-7 cells grown to 90% confluency were transiently transfected with the APP-GFP cDNAs, as described above. After 8 h, cells were divided into six-well plates containing 100 μg/ml polyornithine pre-treated glass coverslips, and left to recover for 4 h. Cells were treated for different times (0, 1, 2, 3 and 5 h) with 50 μg/ml CHX in FBS- and p/s-free DMEM [45] and harvested with 1% SDS. The total protein content of the cellular lysates was determined using a BCA kit (Pierce). Mass-normalized samples were subjected to 6.5% SDS-PAGE in Tris-Glycine buffer, and electrophoretically transferred onto nitrocellulose membranes. Immunoblotting of the transferred proteins was performed by incubating membranes O/N with primary antibodies after blocking non-specific binding sites with non-fat dry milk in TBS-T (10 mM Tris-HCl at pH 8.0, 150 mM NaCl, 0.5% Tween). Detection was achieved using horseradish peroxidase-linked secondary antibodies and an ECL kit (GE Healthcare).

### Wt and S655 mutants APP-GFP lysosomal targeting

COS-7 cells transiently expressing the Wt, S655A or S655E APP-GFP cDNAs were incubated with CHX for 3 h and processed for immunocytochemistry analysis as described above. Upon fixing, cells were processed for immunocytochemistry with the anti-cathepsin D antibody. Cathepsin D is a known lysosomal marker previously used in APP subcellular localization studies [53], which is mainly sorted directly from the TGN to lysosomes. For the chloroquine assay, cells transiently expressing S655A and S655E APP-GFP were exposed to CHX (as described for the APP-GFP turnover rate) and then treated with 50 μM of chloroquine (CQ), (Sigma). This drug is a known inhibitor of lysosomal hydrolases that act by neutralizing lysosomal pH. CQ retards mature APP lysosomal degradation [85]. Cell lysates were collected at the specified CHX time points and subsequently analyzed by immunoblotting using the anti-GFP antibody.

### Secreted Abeta analysis

The medium of COS-7 cells grown on 60 mm plates and transiently expressing the pEGFP vector or the APP-GFP cDNAs was exchanged upon 12 h of transfection for 1.5 ml fresh p/s- and serum-free DMEM. After a 3 h incubation period, this conditioned medium was collected, centrifuged at 310 g for 5 min, and the resultant supernatant immediately frozen in dry ice for Abeta peptides analysis [86]. Media were immunoprecipitated using 25 μl dynabeads (Dynal, Germany) coated with 1E8 mAb. Immunoprecipitates were separated on 12% Bicine/Tris gel containing 8 M urea. Different Abeta peptide species were detected by immunoblotting using mAb 1E8 [87,88]. Total Abeta secretion was calculated by densitometric analysis of all Abeta peptide bands, corrected for holo APP-GFP transfection levels and expressed as fold-increases of Wt total Abeta.

### Immunoprecipitation of APP, VPS35 and SorLA

COS-7 cells, non-transfected or transfected with the APP-GFP cDNAs for 24 h by means of Lipofectamine, were washed with PBS and collected with a scraper in lysis buffer [50 mM TrisHCl ph 8.0, 100 mM NaCl, 1 mM EDTA, 1% CHAPS, containing 0.2 mM PMSF and a protease inhibitor cocktail (Sigma)] and briefly sonicated on ice. Protein mass normalized aliquots were pre-cleared for 1 h with 25 μl protein G sepharose (for VPS35 IP; GE Healthcare) or anti-mouse IgGs agarose beads (for 22C11 or JL-8 IPs; Sigma). Following removal of the beads, lysates were incubated overnight at 4°C with the appropriate primary antibodies, anti-GFP JL-8 (1:200) or 22C11 (1:20) and VPS35 (1:20), and further incubated for 3 h with 50 μl of the appropriated beads pre-cleared for 1 h with 5% BSA. HEK293T cells were transfected with SorLA DNA, APP_695 _and EGFP using Lipod293 reagent (SignaGen). Cells were harvested 48 h following transfection, washed with PBS and lysed in lysis buffer [50 mM TrisHCl ph 7.5, 100 mM NaCl, 1 mM EDTA, 1% Nonidet P-40, 0.2 mM PMSF, 0.2 mM Na_3_VO_4_, 50 mM NaF, 10 mM sodium pyrophosphate, containing a protease inhibitor cocktail (Roche)]. For Immunoprecipitation, the lysates were pre-cleared with the appropriate control IgG and 20 μl of protein A/G agarose (Santa Cruz) for 30 min. Following removal of A/G agarose beads, lysates were incubated with 1 μg of the appropriate primary antibody (anti-SorLA BD transduction labs, or APP C-terminal antibody, 369) or pre-immune serum (negative control) overnight at 4°C, followed by a further 3 h with protein A/G agarose beads.

Following the 3 h incubation period, beads were subsequently washed 4 times with washing buffer (lysis buffer minus CHAPS) or lysis buffer, resuspended in SDS sample buffer and boiled at 95°C for 5 min prior to analysis by SDS PAGE. Western blots were performed using anti-APP (22C11 and 369), anti-VPS35, anti-SorLA (C-terminal antibody) and anti-GFP antibodies.

### VPS35 siRNAs antibody uptake and turnover assays

VPS35 expression was transiently down-regulated using Silencer Select Pre-designed siRNAs (Applied Biosystems/Ambion) against human VPS35 mRNA (GenBank: NM_018206.4, 99% homologous to monkey VPS35 mRNA): s31374: 5'CCACGUUGAUUCAAGAUCAtt; s31375: 5'CCAUGGAUUUUGUACUGCUtt3'; s31376: 5'GUCUGUUUCUUGAAAUUAtt3'. To optimize the siRNA conditions, 5x10^4 ^COS 7 cells were plated on a 24-well plate, 24 hours before being co-transfected with 0.5 μg N1 EGFP cDNA and 0, 1, 5, 10 and 20 nM of each VPS35 siRNA duplex by means of TurboFect siRNA Transfection Reagent (Fermentas Life Science), following the manufactures' instructions. Down-regulation of VPS35 expression was monitored in cells lysates upon 24 h (and 48 h, data not shown) of transfection. To perform the antibody uptake and turnover experiments, 5x10^4 ^COS 7 cells were plated on 24-well plates 24 hours before being co-transfected with 0.5 μg S655E APP-GFP cDNA and 5 nM of each VPS35 siRNA duplex. Upon 24 hours of transfection, cells were incubated in 50 μg/ml CHX for several time points (0, 1, 2, 3, and 5 h), after which they were processed for immunoblotting procedures as described above (holo APP-GFP turnover rate assay). A subset of S655E/siRNAs co-transfected cells, previously grown on polyornithine-coated glass coverslips, was subjected to the above described 22C11 antibody uptake assay upon 2:30 h in 50 μg/ml CHX, with endocytosis being allowed to occur for 15 min at 37°C.

### Protein band quantification and statistical analysis

Autoradiograms were scanned in a GS-710 calibrated imaging densitometer (Bio-Rad) and protein bands quantified using the Quantity One densitometry software (Bio-Rad). Data are expressed as mean ± SEM of at least three independent experiments. Statistical significance analysis was conducted by one way analysis of variance (ANOVA) followed by the Tukey-Kramer test, with the level of statistical significance set at p < 0.05. For the CQ and VPS35 siRNAs APP-GFP turnover rate assays, the two-sided Student t test was used, with statistical significance set at p ≤ 0.05.

### Image acquisition and quantification

Epifluorescence microscopy was carried out using an Olympus IX-81 motorized inverted microscope equipped with Olympus LCPlanFl 20 ×/0.40 and 60 ×/0.70 objective lens. Photographs were taken at 18°C with a Digital CCD monochrome camera F-View II (Soft Imaging System) and processed with the AnalySIS software (Soft Imaging System). For confocal microscopy, images were acquired in a LSM 510 META confocal microscope (Zeiss) using an Argon laser line of 488 nm (APP-GFP channel), a 561 nm DPSS laser (Texas red and Alexa Fluor 568 labels channel), and a Diode 405-430 laser (ECFP and Alexa Fluor 350 labels channel). Quantitative correlation analysis (e.g., [89,90]), was carried out with the Zeiss LSM 510 4.0 software, using images of all cells populations (endogenous APP observations) or images of delimited single cells (APP-GFP transfected cells). For co-localization of Wt APP-GFP with protein markers of the endocytic pathway, the co-localization coefficients were determined as the percentage of APP-GFP/Transferrin, APP-GFP/Rab 5 and APP-GFP/Rab 7 co-localizing pixels relatively to the number of pixels in the APP-GFP channel. For lysosomal targeting, the co-localization coefficients were determined as the percentage of APP-GFP/cathepsin D co-localizing pixels relatively to the number of pixels in the APP-GFP channel. For endocytosed APP-GFP targeted to the TGN, the co-localization coefficients were determined as the percentage of Texas red-22C11/APP-GFP co-localizing pixels at the Golgi relatively to the number of pixels in the Texas red-22C11 channel. For retromer co-localization, the co-localization coefficients were determined as the percentage of Alexa350-AU 22C11 or APP-GFP/VPS35 co-localizing pixels relatively to the number of pixels in the Alexa350-AU 22C11 or APP-GFP channels, respectively.

## Abbreviations

AD: Alzheimer's disease; APP: amyloid precursor protein; AU: antibody uptake; CatD: Cathepsin D; CHX: cycloheximide; CFP: cyan fluorescent protein: CQ: Chloroquine; GFP: green fluorescence protein; IP: immunoprecipitation; p/s: penicillin/streptomycin; PKC: protein kinase C; PM: plasma membrane; siRNA: small interfering RNA; TGN: trans-Golgi network; VPS: vacuolar protein sorting; WB: western blot.

## Competing interests

The authors declare that they have no competing interests.

## Authors' contributions

All authors have contributed for the preparation of this manuscript and have read and approved the final manuscript.

## Supplementary Material

Additional file 1 **Retrieval of clathrin/APP-GFP endocytic vesicles from endosomes to the TGN is hindered at 19.5°C**. Wt APP-GFP expressing COS-7 cells were subject to the previous 22C11 uptake assay (Alexa350 blue staining). Following 15 min at 19.5°C, Clathrin/APP-GFP endocytic vesicles can still be observed, with a good juxtaposed co-localization. Further, these vesicles appear more dispersed and the number of tubulating vesicles is clearly diminished. ROI, region of interest, 2.0-fold magnified. Bar, 10 μm. Click here for file

Additional file 2 **Redistribution of APP and VPS35 proteins upon exposure of HeLa cells to PDBu**. HeLa cells were cultured in MEM medium supplemented with Glutamax and 10% FBS (Gibco BRL), and exposed for two hours to 1 μM PDBu, a known PKC inducer. Cells were fixed and subjected to immunocytochemistry procedures using an anti-APP C-terminus antibody (green FITC secondary labeling) and an antibody against the retromer component VPS35 (red Alexa Fluor 568 secondary labeling). Cell nuclei were stained with DAPI (blue fluorescence). Redistributions of the protein populations, from a more cytoplasmic diffuse morphology to a more perinuclear Golgi-like concentrated pattern (arrows), can be observed in response to PDBu. Bar, 10 μm. Click here for file

Additional file 3 **Representative profile of APP-GFP C-terminal peptides with time in CHX**. Immunoblot analysis (12% SDS-PAGE) of Wt APP-GFP transfected COS-7 cells lysates using the anti-GFP JL-8 antibody. The bands around and below ~37 kDa correspond to APP-GFP C-terminal peptides (CTPs-GFP), positive for the anti-APP C-terminal antibody and negative for 22C11 against APP N-terminus (data not shown). S655A and S655E mutants render similar CTPs-GFP profiles when in CHX (data not shown). Top and bottom panels correspond to cropped areas of the same autoradiogram, and therefore have the same exposure time conditions. Click here for file

Additional file 4 **Omission of cell permeabilization in the 22C11 uptake assay impairs visualization of APP-GFP endocytic vesicles**. COS-7 cells expressing the Wt, S655A and S655E APP-GFP proteins were pre-incubated at 4°C to inhibit endocytosis (0 min). Addition of the 22C11 anti-APP ectodomain antibody ("APP N-term"), allowed for the labelling of APP-GFP proteins at the cell surface. At 0, 15, and 30 min of incubation at 37°C, cells were subjected to immunocytochemistry procedures with a Texas red secondary antibody without previous permeabilization. Clear endocytic vesicles (e.g. as observed in Fig. 3 and 5) were no longer visible when cell permeabilization is omitted. Instead, a surface dot-like staining could be observed for the 22C11 antibody (0 min), which decreased with time of 37°C incubation, in accordance with continuous 22C11/APP-GFP surface clearance by endocytosis. ROI, region of interest. Bar, 10 μm. Click here for file
