# Supplementary figures and images for: Retrieval of the Alzheimer's amyloid precursor protein from the endosome to the TGN is S655 phosphorylation state-dependent and retromer-mediated
Source: Mol Neurodegener. 2010 Oct 11;5:40. doi: 10.1186/1750-1326-5-40 (PMC2994555; doi:10.1186/1750-1326-5-40)

Wt APP-GFP

22C11 Uptake

Clathrin

Overlay

15 min 19.5°C

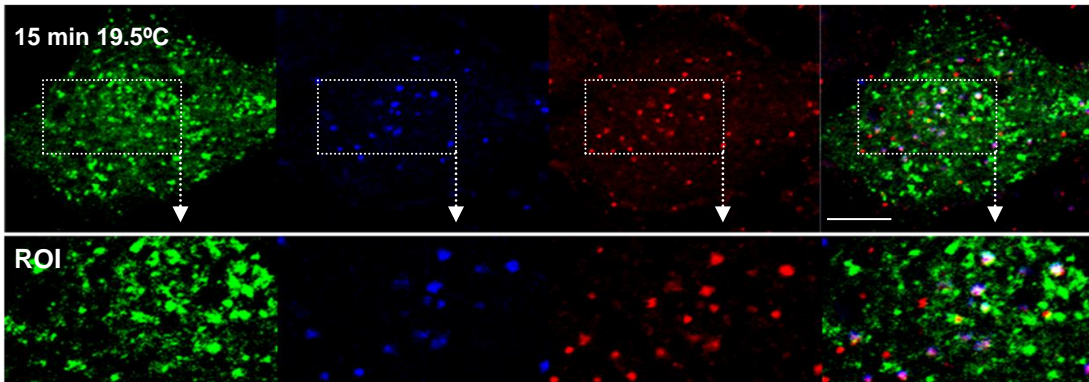

Supplement: Additional file 1 — Retrieval of clathrin/APP-GFP endocytic vesicles from endosomes to the TGN is hindered at 19.5°C. Wt APP-GFP expressing COS-7 cells were subject to the previous 22C11 uptake assay (Alexa350 blue staining). Following 15 min at 19.5°C, Clathrin/APP-GFP endocytic vesicles can still be observed, with a good juxtaposed co-localization. Further, these vesicles appear more dispersed and the number of tubulating vesicles is clearly diminished. ROI, region of interest, 2.0-fold magnified. Bar, 10 μm. [file 1750-1326-5-40-S1.PDF]

- PDBu

+ PDBu

DAPI

DAPI

VPS35

VPS35

APP

APP

Overlay

Overlay

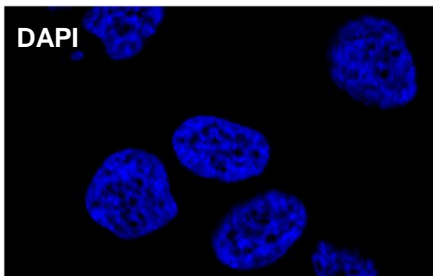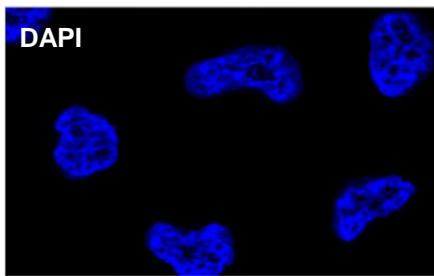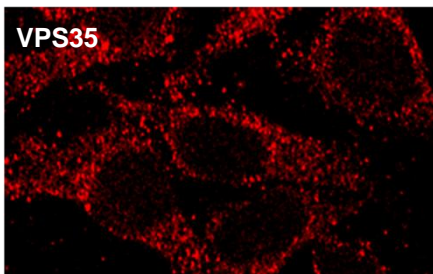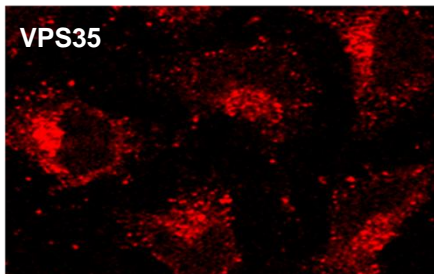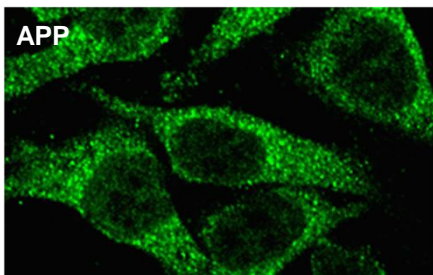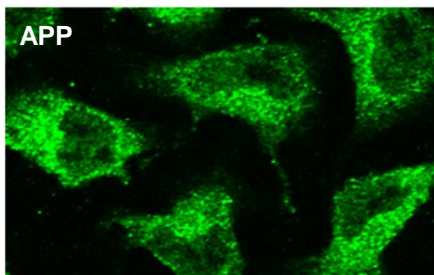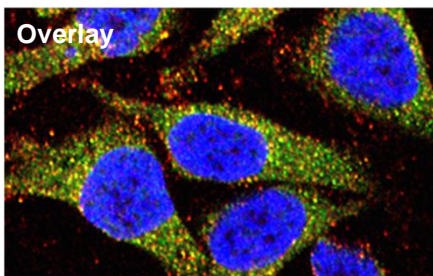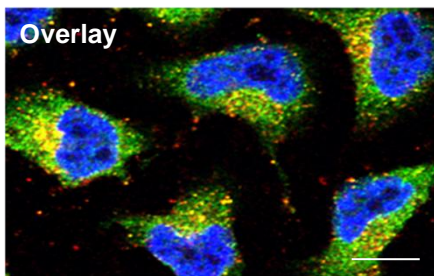

Supplement: Additional file 2 — Redistribution of APP and VPS35 proteins upon exposure of HeLa cells to PDBu. HeLa cells were cultured in MEM medium supplemented with Glutamax and 10% FBS (Gibco BRL), and exposed for two hours to 1 μM PDBu, a known PKC inducer. Cells were fixed and subjected to immunocytochemistry procedures using an anti-APP C-terminus antibody (green FITC secondary labeling) and an antibody against the retromer component VPS35 (red Alexa Fluor 568 secondary labeling). Cell nuclei were stained with DAPI (blue fluorescence). Redistributions of the protein populations, from a more cytoplasmic diffuse morphology to a more perinuclear Golgi-like concentrated pattern (arrows), can be observed in response to PDBu. Bar, 10 μm. [file 1750-1326-5-40-S2.PDF]

CHX h:

0

1

2

3

5

**Wt APP-GFP**

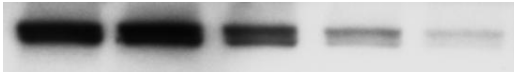

- 150

**CTPsGFP →**

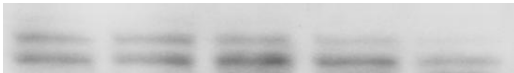

- 37

Supplement: Additional file 3 — Representative profile of APP-GFP C-terminal peptides with time in CHX. Immunoblot analysis (12% SDS-PAGE) of Wt APP-GFP transfected COS-7 cells lysates using the anti-GFP JL-8 antibody. The bands around and below ~37 kDa correspond to APP-GFP C-terminal peptides (CTPs-GFP), positive for the anti-APP C-terminal antibody and negative for 22C11 against APP N-terminus (data not shown). S655A and S655E mutants render similar CTPs-GFP profiles when in CHX (data not shown). Top and bottom panels correspond to cropped areas of the same autoradiogram, and therefore have the same exposure time conditions. [file 1750-1326-5-40-S3.PDF]

### 0 min w/o permeabilization

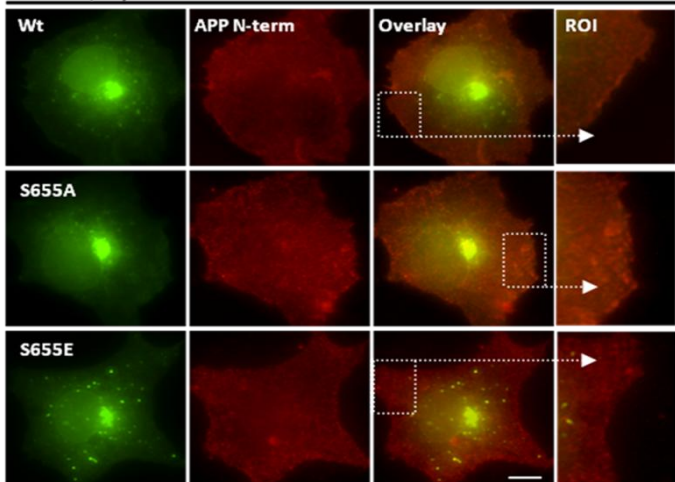

### 15 min w/o permeabilization

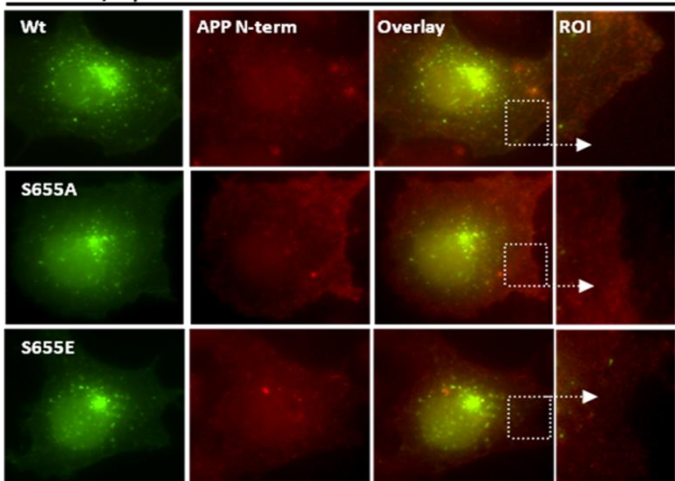

### 30 min w/o permeabilization

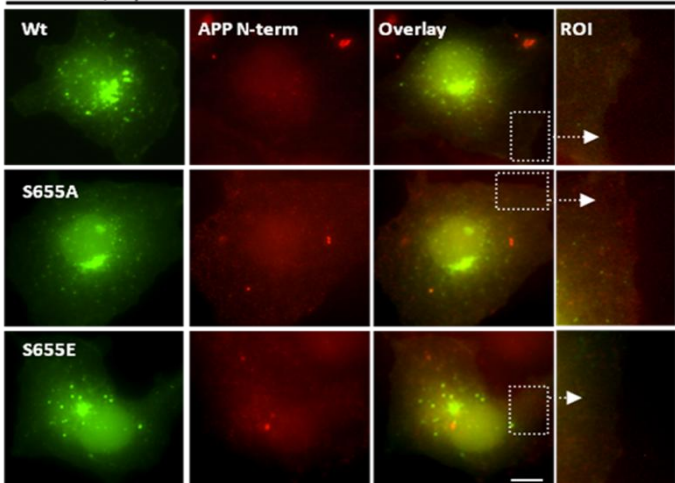

Supplement: Additional file 4 — Omission of cell permeabilization in the 22C11 uptake assay impairs visualization of APP-GFP endocytic vesicles. COS-7 cells expressing the Wt, S655A and S655E APP-GFP proteins were pre-incubated at 4°C to inhibit endocytosis (0 min). Addition of the 22C11 anti-APP ectodomain antibody ("APP N-term"), allowed for the labelling of APP-GFP proteins at the cell surface. At 0, 15, and 30 min of incubation at 37°C, cells were subjected to immunocytochemistry procedures with a Texas red secondary antibody without previous permeabilization. Clear endocytic vesicles (e.g. as observed in Fig. 3 and 5) were no longer visible when cell permeabilization is omitted. Instead, a surface dot-like staining could be observed for the 22C11 antibody (0 min), which decreased with time of 37°C incubation, in accordance with continuous 22C11/APP-GFP surface clearance by endocytosis. ROI, region of interest. Bar, 10 μm. [file 1750-1326-5-40-S4.PDF]
